# Feasibility of Modified Surviving Sepsis Campaign Guidelines in a Resource-Restricted Setting Based on a Cohort Study of Severe *S. Aureus* Sepsis

**DOI:** 10.1371/journal.pone.0029858

**Published:** 2012-02-21

**Authors:** Weera Mahavanakul, Emma K. Nickerson, Pramot Srisomang, Prapit Teparrukkul, Pichet Lorvinitnun, Mingkwan Wongyingsinn, Wirongrong Chierakul, Maliwan Hongsuwan, T. Eoin West, Nicholas P. Day, Direk Limmathurotsakul, Sharon J. Peacock

**Affiliations:** 1 Department of Medicine, Sappasithiprasong Hospital, Ubon Ratchathani, Thailand; 2 Mahidol-Oxford Tropical Medicine Research Unit, Faculty of Tropical Medicine, Bangkok, Thailand; 3 Department of Medicine, University of Cambridge, Addenbrooke's Hospital, Cambridge, United Kingdom; 4 Department of Pediatrics, Sappasithiprasong Hospital, Ubon Ratchathani, Thailand; 5 Department of Anesthesiology, Faculty of Medicine, Siriraj Hospital, Bangkok, Thailand; 6 Department of Tropical Medicine, Faculty of Tropical Medicine, Mahidol University, Bangkok, Thailand; 7 Department of Medicine and the International Respiratory and Severe Illness Center, Harborview Medical Center, University of Washington, Seattle, Washington, United States of America; 8 Centre for Clinical Vaccinology and Tropical Medicine, Nuffield Department of Clinical Medicine, University of Oxford, Oxford, United Kingdom; 9 Department of Tropical Hygiene, Faculty of Tropical Medicine, Mahidol University, Bangkok, Thailand; 10 Department of Microbiology and Immunology, Faculty of Tropical Medicine, Mahidol University, Bangkok, Thailand; Institut Pasteur, France

## Abstract

**Background:**

The Surviving Sepsis Campaign (SSC) guidelines describe best practice for the management of severe sepsis and septic shock in developed countries, but most deaths from sepsis occur where healthcare is not sufficiently resourced to implement them. Our objective was to define the feasibility and basis for modified guidelines in a resource-restricted setting.

**Methods and Findings:**

We undertook a detailed assessment of sepsis management in a prospective cohort of patients with severe sepsis caused by a single pathogen in a 1,100-bed hospital in lower-middle income Thailand. We compared their management with the SSC guidelines to identify care bundles based on existing capabilities or additional activities that could be undertaken at zero or low cost. We identified 72 patients with severe sepsis or septic shock associated with *S. aureus* bacteraemia, 38 (53%) of who died within 28 days. One third of patients were treated in intensive care units (ICUs). Numerous interventions described by the SSC guidelines fell within existing capabilities, but their implementation was highly variable. Care available to patients on general wards covered the fundamental principles of sepsis management, including non-invasive patient monitoring, antimicrobial administration and intravenous fluid resuscitation. We described two additive care bundles, one for general wards and the second for ICUs, that if consistently performed would be predicted to improve outcome from severe sepsis.

**Conclusion:**

It is feasible to implement modified sepsis guidelines that are scaled to resource availability, and that could save lives prior to the publication of international guidelines for developing countries.

## Introduction

Sepsis is the systemic inflammatory response to infection [1], [2]. Severe sepsis and septic shock are estimated to affect millions of people each year and are important causes of mortality worldwide [3], [4], [5], [6]. The Surviving Sepsis Campaign (SSC) guidelines first published in 2004 [7] and updated in 2008 [8] are highly influential documents that describe best practice for the management of sepsis in resource-rich settings, and represent a milestone in the improvement of clinical care standards in the developed world. However, the majority of sepsis deaths worldwide occur in the developing world, where bacterial infections are implicated as the direct cause of death from lower respiratory tract infections, meningitis, and a range of other infections [9], [10], [11], [12] and also complicate other common diseases such as malaria, HIV/AIDS and diabetes mellitus [13], [14], [15]. Severe sepsis in resource-limited low- and middle-income countries is understudied but is associated with extremely high mortality rates [16], [17], [18], [19], yet with the exception of recent pandemic influenza guidelines [20] no recommendations exist that detail effective approaches to sepsis care in these settings [21].

The facilities and therapeutics available for the management of patients with sepsis beyond the developed world are highly variable and form a wide spectrum of capabilities, including a developing intensive care service in many under-resourced healthcare settings [22]. We have proposed previously that modified guidelines are required to promote a scaled approach to management based on optimal utilisation of existing resources [23]. Here, we describe a prospective observational study of patients with severe sepsis treated at a hospital in provincial, northeast Thailand where the SSC guidelines have not been implemented because of resource limitations. We determined the extent to which these guidelines are already incorporated into patient care, identified the range of variability in their application, and designed achievable care bundles consisting of activities that could be implemented within available capabilities resources and without delay.

## Methods

### Ethical statement

The Ethics Committee of the Ministry of Public Health, Royal Government of Thailand and the Oxford Tropical Research Ethics Committee approved the study.

### Setting and facilities

Sappasithiprasong Hospital is situated in the provincial town of Ubon Ratchathani in northeast Thailand, 70 km west of Laos and 95 km north of Cambodia. This 1,100-bed hospital serves around 2 million people, the majority of whom are rice farmers. Facilities include diagnostic laboratory and radiology services including a CT scanner. General medical, surgical and orthopaedic wards are mainly open-plan with around 30 beds per ward, increasing to 60 beds per ward in response to demand. There are 13 separate intensive care units (ICU) (four paediatric and nine adult), each containing between eight and 14 beds. Piped oxygen is available in each ICU for all beds, along with bedside monitoring for oxygen saturations. On the general wards, oxygen is piped to the half of the ward closest to the nurses' area, with tanks of oxygen available for the remainder of the beds. Bedside monitoring for oxygen saturations is available for four to five beds per general ward, with one to two portable pulse oximeters available per ward for the remainder. Full blood count, renal and liver function tests, plasma glucose and coagulation tests are available, while plasma lactate, plasma C-reactive protein and procalcitonin are not available. Disposable peripheral intravenous devices are available throughout the hospital. Red blood cell transfusions are obtainable, but supplies are limited and family members may be expected to donate blood to facilitate transfusions, particularly with uncommon blood groups. Use of standard central venous devices is limited to ICUs, with the use of cut-downs inserted at the antecubital fossa for central venous access in general wards. Electrically powered multi-function ventilators (e.g. Bear Cub, Infant Star, or Bennett) are available in ICUs, while patients on general wards may be ventilated by older gas driven ventilators (Bird Mark 7). Haemofiltration machines are available but generally restricted to treatment of patients with chronic renal failure because of limited resources. All of the major classes of parenteral antimicrobial agents are available including the carbapenems. The registered nurse-to-patient ratio is approximately 1∶8 on general wards and 1∶2.5–3.5 on ICUs. The workload of attending physicians is extremely high and timing of attendance to new and existing patients is prioritised based on an assessment of severity of illness made by healthcare attendants, including medical students and interns.

### Patient recruitment

Our objective was to identify patients with severe sepsis due to a single, common human pathogen, and chose *Staphylococcus aureus* as it represents a global pathogen and is the third most common blood culture isolate at Sappasithiprasong Hospital (after *Burkholderia pseudomallei* and *Escherichia coli*). All patients with one or more clinically significant blood cultures positive for *S. aureus* were identified over a period of one year (November 2006 to November 2007). Cases were identified each day at the diagnostic microbiology laboratory, followed by same day ward visit. Patients were recruited after obtaining written informed consent and visited daily until discharge. Detailed clinical information including patient management was recorded from the patient chart using standardised forms for the three day period starting from the day the first culture positive for *S. aureus* was taken (to reflect the 72 hours of early goal-directed therapy for sepsis described by Rivers et al) [24]. Outcome (survival or death) was defined 28 days after the first positive culture was taken, with phone call follow up for those patients discharged from hospital alive. The cases described here represent a subset of a larger cohort reported previously [25], [26].

### Defining sepsis

Children were defined as 18 years of age or less to be consistent with the paediatric sepsis classification [27] (patients aged 14 years or more are treated on adult wards in our setting). Data from the first 24 hours after the first positive culture was taken were used to categorise patients as having sepsis, severe sepsis and septic shock. Published definitions of sepsis [1], [2], [27] were used, as follows: (i) sepsis was defined as systemic inflammatory response syndrome (SIRS) associated with infection; (ii) severe sepsis was SIRS with organ dysfunction, arterial hypotension, or tissue hypoperfusion as denoted by acidosis or clinical signs; (iii) septic shock was SIRS with arterial hypotension. Defining septic shock as hypotension despite adequate fluid resuscitation was not possible because recording of fluid volumes given to study patients was incomplete. Diagnostic criteria for organ dysfunction were modified from the SSC guidelines to classify patients based on available data (Table 1).

**Table 1:** Diagnostic criteria used for organ dysfunction.

Organ system	Organ dysfunction variables
Kidney	Acute oliguriaUrine output <500 ml per 24 hrs or<12 ml/kg per 24 hrs in paediatric patients
	AzotaemiaCreatinine >2 mg/dl (177 µmol/l) or>2 times upper limit of normal for age
Haematologic	ThrombocytopaeniaPlatelets <100×10^9^/l or<80×10^9^/l in paediatric patients
Liver	Hyperbilirubinaemia>2 mg/dl (34.2 µmol/l) in adults, or >4 mg/dl (except newborns), or ALT >2 times upper limit of normal for age in paediatric patients
Respiratory	Mechanical ventilation
Cardiovascular	Arterial hypotensionSBP <90 mmHg, or <age-specific BP in paediatric patients (septic shock), or use of vasoactive drugs (dopamine only >5 µg/kg/min)

Criteria were from the literature [1], [2], [27] to fit the available data. Acute oliguria was determined from 24-hour urine because hourly urine output was infrequently monitored. Diagnostic criteria of arterial hypoxaemia (PaO_2_/FiO_2_<300), ileus, and clinical signs of tissue hypoperfusion (decrease capillary refill or mottling) were not used as data were not recorded in the patient records. Laboratory testing for lactate level was not available in the hospital. The Glasgow Coma Score was not documented in patient records.

### Evaluation of sepsis management

The details of patient management during the three days after the first positive culture was taken were recorded with particular reference to table 3 (initial resuscitation and infection issues), table 4 (haemodynamic support and adjunctive therapy), and table 5 (other supportive therapy of severe sepsis) contained within the 2008 SSC Campaign guidelines [8]. Recommended activities that were undertaken during routine clinical care in our hospital were noted, together with the proportion of patients who received each activity. Using these data, we then designed a sepsis management programme based on activities that were already part of routine care, together with activities that were not routine that fell within existing resources or could be implemented at zero or very low additional cost. We described two care bundles, one for use on general wards (bundle I) and the second for use on intensive care units (bundle II).

## Results

### Identification of patients with severe sepsis

**Figure 1:**
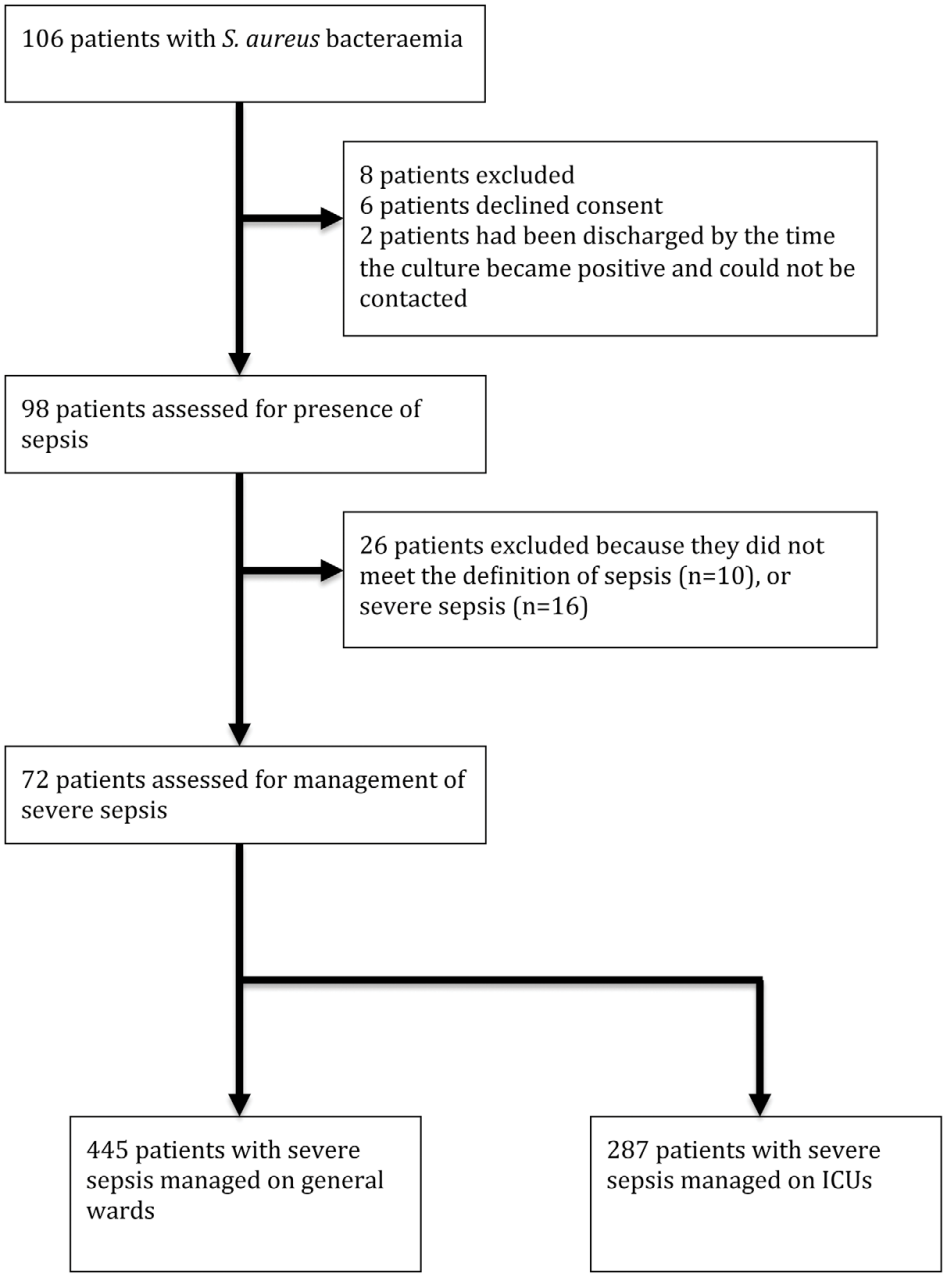
Study flow diagram.

We identified a total of 106 eligible patients with *S. aureus* bacteraemia, of whom 98 were recruited (Figure 1). Of the 98 evaluable patients, 57 (58%) were male, and age ranged from one day to 92 years (median 39 years, interquartile range 9 to 65 years). A history of any chronic underlying medical condition was documented in 57 cases (58%), with cardiac disease accounting for the greatest proportion (19 cases, 19%). An identified site of infection (beyond bacteraemia) was found in 59 patients (60%). Methicillin-resistant *S. aureus* (MRSA) was responsible for infection in 27 patients (28%). Vital signs and full blood count were performed in every patient. From this we determined that 88 of the 98 patients (90%) with *S. aureus* bacteraemia met the criteria for sepsis within the three day period starting on the day that the positive blood culture was taken. Of these 88 patients, 72 (49 adults) had severe sepsis, including a subset of 48 patients (31 adults) with septic shock.

### Management of 72 patients with severe sepsis

Of the 72 patients with severe sepsis, 28 patients (14/23 children [61%] and 14/49 adults [29%]) received care in an ICU and the remainder were treated on a general ward. The adult ICUs ran at full occupancy during the study period and access was strongly influenced by bed availability. Their sepsis management is summarised in Table 2. The 28 day mortality rate was 53% (38/72 patients), and half of the patients who died did so within 3 days after the day the first culture positive for *S. aureus* was taken. The mortality for patients with septic shock was 60% (29/48 patients) versus 38% (9/24 patients) for those not in septic shock; and the mortality for patients cared for in an ICU was 61% (17/28 patients) versus 48% (21/44 patients) for general ward based care.

**Table 2:** Management and outcome of 72 patients with severe sepsis.

	Number of patients (%)
**Place of care**	
ICU	28 (39%)
General wards	44 (61%)
**Fluid resuscitation**	
Use of crystalloid fluid	72 (100%)
Documented fluid bolus	19 (26%)
Monitor and goal of fluid resuscitation	
Record fluid balance	52 (72%)
Urinary catheterisation	37 (51%)
Central venous access	12 (17%)
Record CVP	6 (8%)
**Antibiotic therapy**	
Broad-spectrum intravenous antibiotic before culture result	68 (94%)
Broad-spectrum intravenous antibiotic effective against infecting organism	53/68 (78%)
Effective antibiotic given after culture result	48/49 (98%)
**Source identification and control**	
Any radiological imaging	62 (86%)
Chest radiography	55 (76%)
Identify anatomical site of infection	46 (64%)
Use procedure to control infectious source where applicable	15/15 (100%)
**Respiratory support**	
Supplementary oxygen if not ventilated	20/36 (55%)
Mechanical ventilation	36 (50%)
Monitor oxygen level	
With oxygen saturation alone	26 (36%)
With arterial blood gas (at least one value)	15 (21%)
**Vasopressor and inotropic therapy**	
Vasoactive drug use in patients with septic shock	26/48 (54%)
Dobutamine use in patients with septic shock	7/48 (15%)
**Other support**	
Blood product administration in patients with haemoglobin less than 7.0 g/dl	7/10 (70%)
Subcutaneous insulin to control hyperglycaemia	8 (11%)
**Outcome**	
28 day mortality	38 (53%)
28 day mortality in subset with septic shock	29/48 (60%)
28 day mortality in subset managed on ICU	17/28 (61%)
28 day mortality in subset managed on general ward	21/44 (48%)

When not shown the patient denominator is 72. Where the denominator differs from this for a particular question, these are shown.

The timing of severe sepsis recognition and initial resuscitation measures were variably documented in patient records. Crystalloid was used as initial fluid resuscitation in all cases, and a blood pressure of 90/60 mmHg was generally used as a goal of the resuscitation. Fluid challenges were documented in 19 patients (26%), all of whom had septic shock. The initial challenge volume ranged from 150 to 250 mls. The total volumes of fluid infused were not recorded. A central venous device was placed in 12 patients (17%; 8 were cut-downs), of whom 6 patients (all with septic shock) had central venous pressure (CVP) documented. Invasive monitoring of arterial pressure via an intra-arterial catheter was only possible in the cardiothoracic wards, and was not performed on any of the 5 study patients on this ward. A urinary catheter was inserted in 37 patients (51%), and urinary output was recorded in 43 patients (60%). Study eligibility criteria meant that all study patients had at least one blood culture taken; ≥2 blood cultures were taken from 10 patients (14%) during initial assessment. The majority of patients (86%) underwent radiological imaging, with 55 patients (76%) having a chest radiograph, 15 patients (21%) an ultrasound or Doppler scan, and 8 patients (11%) a computed tomography (CT) scan.

A broad-spectrum intravenous antibiotic was received by 68 patients (94%) within 24 hours after the first positive blood culture was taken, although the time of administration of the first antibiotic dose was not recorded. The infecting isolate was susceptible to the empiric antimicrobial drug used in 53 cases, but not in 15 cases (all infected with MRSA). Once culture results were available, almost all patients who were still alive (48 out of 49, 98%) received an antibiotic that covered their infecting isolate of *S. aureus*. A specific anatomical site of infection was identified in 46 patients, of whom 15 patients had a procedure contributing to infectious source control (abscess drainage 6, debridement 5, joint wash-out 1, fasciotomy 1, above knee amputation 1, chest drain insertion 1). A further 5 patients had a potentially drainable collection of pus identified or infected prosthetic material but did not undergo a procedure as this was deemed unfeasible because of the presence of a coagulopathy, or clinical instability that prevented movement of the patient to another department.

One or more vasoactive drugs were used in 26 out of 48 patients with septic shock (54%), dopamine being used most often as the first line agent. The majority of patients prescribed vasopressors or inotropes (65%) received the drug through a peripheral intravenous catheter. Steroids were not used in the management of sepsis, and recombinant human activated protein C (rhAPC) was not available. Red blood cell transfusions were given in 7 of 10 patients (70%) with haemoglobin less than 7.0 g/dl, and were targeted to reach a haemoglobin of ≥9 mg/dl.

Supplemental oxygen was given to 39 patients (54%), of whom 11 (28%) were monitored by arterial blood gases and 15 (35%) had at least one oxygen saturation result recorded in their notes. Specific data on levels of hypoxaemia were not recorded as part of the study, and it was not possible to calculate the inspired fraction of oxygen as oxygen delivery was not controlled/quantified. A total of 36 patients (50%) were ventilated. The Bird Mark 7 model was used to ventilate 22 patients (16 on general wards and 6 in ICUs), of whom 2 (9%) survived (both in an ICU). Electrically powered mechanical ventilators were used in 14 patients (all in ICUs), of whom 4 (29%) survived. Sedation was variably given to ventilated children, but was not routine for ventilated adults.

Glucose control was achieved by intermittent point-of-care testing of capillary blood glucose and subcutaneous insulin. None of the 18 patients with acute renal failure or an additional 15 patients who were acidotic received dialysis. No patients received deep vein thrombosis or stress ulcer prophylaxis.

### Modified care bundles for severe sepsis management

Based on resources available to us together with information from the published literature, we devised two incremental care bundles for patients presenting with suspected infection in our setting (Table 3, 4 and 5). Bundle I is for use on general wards and includes interventions that are already performed in some cases but that could be implemented in all cases using available resources through education of healthcare workers and at no or modest increase in costs. Central to the diagnosis of severe sepsis is assessment of organ dysfunction. This can be accomplished by expanding the clinical assessment of the patient with suspected infection (evaluating and recording Glasgow Coma Scores, capillary refill (in children), calculating PaO2 or SpO2 and inspired oxygen levels for those on supplemental oxygen) and measuring serum creatinine and bilirubin in addition to the full blood count [28]. The critical components of management are administration of antibiotics within 1 hour of recognition of sepsis, and provision of adequate volumes of intravenous fluid with respiratory/haemodynamic support to maintain tissue oxygen delivery, using an iterative resuscitation strategy. Point of care lactate measurement is a simple and useful tool both to diagnose and manage severe sepsis that is not readily available currently but that we propose is a worthwhile investment [29], [30]. Bundle II includes a combination of ICU-specific interventions that require no additional resources and that could be implemented immediately (e.g. PEEP in mechanically ventilated patients with respiratory failure), and those with cost implications (e.g. haemodialysis).

**Table 3:** Initial resuscitation and infection issues in a resource-limited setting.

Recommendation	Bundle I: General ward setting	Bundle II: ICU setting
**Initial evaluation and detection of severe sepsis in patients with suspected infection**	• Determine Glasgow Coma Score, capillary refill	• Admit patients with severe sepsis to ICU
	• Measure PaO_2_/FiO_2_ or SpO2/FiO_2_	• Perform arterial blood gas and calculate PaO_2_/FiO_2_
	• Measure serum creatinine and total bilirubin in addition to full blood count	
	• Use point-of-care test to determine lactate level	
**Fluid resuscitation**	• Give first iv fluid challenge of 1,000 mL (adults) or 20 ml/kg (children) of crystalloids over 30 minutes	• Obtain central venous access
	• Use a combination of mean arterial blood pressure, urine output, and POC lactate reduction as resuscitation goals	• Include CVP and central venous oxygen saturation as goal of resuscitation
**Diagnosis**	• Obtain two or more blood cultures, at least one from venous puncture and one blood culture from each vascular access device in place for more than 48 hrs	
**Antibiotic therapy, and source identification and control**	• Begin intravenous antibiotics within the first hour of recognising severe sepsis	

**Table 4:** Hemodynamic support and adjunctive therapy of severe sepsis in a resource-limited setting.

Recommendation	Bundle I: General ward setting	Bundle II: ICU setting
**Fluid resuscitation**	• Insert a urinary catheter and monitor urine output every 2 hours	• Monitor hourly urine output
	• Use an iterative fluid challenge/clinical response technique	
**Vasopressors and inotropic therapy**	• Administer dopamine peripherally if patient has hypotension despite meeting fluid resuscitation goals	• Administer dopamine centrally if patient has hypotension
		• Use dobutamine in patients with low cardiac filling and low cardiac output
**Steroids**	• Consider iv hydrocortisone for adults with septic shock when hypotension responds poorly to adequate fluid resuscitation and vasopressors	

**Table 5:** Other supportive therapy in a resource-limited setting.

Recommendation	Bundle I: General ward setting	Bundle II: ICU setting
**Respiratory support**	• Monitor oxygen saturation	
	• Provide supplemental oxygen, if hypoxaemic	
**Mechanical ventilation**		• Target a tidal volume of 6 ml/kg and an initial upper limit plateau pressure ≤30 cm H_2_0 if acute lung injury (and able to monitor acid base and oxygenation).
		• Maintain mechanical ventilated patients with head of the bed raised to 45°
		• Use sedation protocol
**Glucose control**	• Use intermittent subcutaneous or intramuscular insulin	• Use continuous intravenous insulin
	• Use insulin protocol	
	• Goal of blood glucose <150 mg/dl	
**Blood product administration**	• Give red blood cells when haemoglobin <7.0 g/dl	
**Renal replacement**	• Use haemodialysis, if possible	
**DVT and stress ulcer prophylaxis**	• Use low-dose unfractionated heparin (UFH) or low molecular weight heparin (LMWH)	
	• Use Histamine 2 receptor blocker	

## Discussion

This study of severe staphylococcal sepsis is among the first to quantify the management and outcome of patients with sepsis admitted to a large district general hospital in a lower-middle income country. We consider it highly likely that our facilities and care provision parallels that of many hospitals throughout the world. Existing capabilities were such that the fundamental principles of sepsis management could be achieved, but the current SSC guidelines were not implementable. The need for feasible sepsis management guidelines was clearly demonstrated by the mortality rate of 53% of patients in this study. This contrasts with a mortality rate of 20–29% for septic patients with hypoperfusion or organ dysfunction in the US [31], [32], and 33–52% for severe sepsis patients in Brazil [17]. This prompted us to develop two modified sepsis care bundles. Bundle I focuses on care that is not routine but that can be accomplished with little additional cost and could be implemented even in general ward settings, whereas Bundle II is ICU-specific and the interventions may be more expensive. Extrapolating from published data in high resource settings, the adoption of these bundles would be predicted to be associated with a significant increase in patient survival [33], [34].

Early detection of severe sepsis could be one of the major keys to reducing the high mortality rate of severe sepsis in developing countries. We found that nearly all methods to detect severe sepsis are available in our setting, and could be performed more consistently in patients with suspected infection. As summarised in care bundle I, to capture the organ dysfunction indicative of severe sepsis requires simple additional clinical assessments and routine measures of serum creatinine and bilirubin in addition to the full blood count. We suggest that addition of point-of-care lactate level would be safe, inexpensive and very beneficial to early detection of severe sepsis in our setting.

Many of the existing recommendations for treatment of severe sepsis could also be implemented with little additional expense. We found that the hospital is fully equipped with all material resources required for the fundamentals of sepsis management, including microbiological investigation, fluid resuscitation, antimicrobial drugs, and vasoactive agents. Bundle I specifies many of these interventions, while bundle 2 recognised areas of improvement relating to more complex sepsis management therapies in the ICU such as invasive haemodynamic monitoring, ventilation and dialysis, where variability in practice may relate to a combination of lack of guidelines and resource management. For example, haemodialysis was available but reserved for patients with chronic renal failure, and reassessment of the utilisation of this treatment modality could result in the provision of equipment for a limited number of patients with acute renal failure. Evidence supports the use of haemodialysis for sepsis-related renal failure in low resource settings [35].

Mechanical ventilation also deserves careful consideration. Physicians are often reticent to use currently recommended fluid challenge volumes for fear of fluid overload in the absence of respiratory support, but respiratory failure may in fact be due to incipient lung injury. This study identified that ventilation of 16 patients on a general ward using a Bird Mark 7 model was not associated with a survival benefit (all 16 patients died). These patients were not sedated, and the process by which the efficiency of ventilation was monitored was sub-optimal. This requires an evaluation to either improve ventilation protocol in general wards, or limit the use of ventilators to an ICU setting.

Invasive haemodynamic monitoring is performed routinely in high resource settings but central venous catheters carry mechanical, thrombotic, and infectious complications [36]. Furthermore, how well such catheters indicate adequacy of resuscitation is subject to debate. Thus the balance of benefit versus risk needs to be addressed for each patient in low resource settings. The finding that serial lactate measures are a suitable alternative to ScvO2 monitoring in sepsis management [30] provides additional support for use of point-of-care lactate testing, perhaps in place of targeting a goal of central venous catheter placement in all patients with severe sepsis.

We understand the limitation of human resources versus the time required to implement the recommendations of the bundles described by this study, which could represent high additional cost if more nurses and doctors are required. However, if early detection and early management of sepsis is aggressively undertaken, fewer invasive and time consuming interventions such as haemodialysis and mechanical ventilation due to multiple organ failure will be required. In addition, more lives can be saved.

Some of the limitations of our study are that it focused on a single aetiology of sepsis, was conducted in a single centre, and that data recorded was incomplete. Nonetheless, our findings and proposed bundles of care are likely to be applicable to all-cause sepsis, and it adds to the almost non-existent literature on management and outcomes of severe sepsis in low resource settings.

We propose that the stepped care bundles described here may have utility worldwide. Minor points in both bundles could be individualised, following local surveys of current practice and resources. Many hospitals in low-middle income countries may not have adopted the surviving sepsis campaign because they consider that resources are not available to follow all of the recommendations in the guidelines. However, many hospitals may be able to substantially improve sepsis care and patient outcomes by more consistent or appropriate use of the resources available. Improved management of severely septic patients in low resource settings could impact on millions of lives per year. In the absence of data specific to these settings, our care bundles extrapolate judiciously from studies performed in high resource regions. While this approach seems practical and inaction is ethically indefensible, recently published evidence underscores the necessity of performing research directly in low resource settings [37]. Perhaps the greatest source of uncertainty for clinicians in low resource settings surrounds the relative benefits of aggressive fluid challenges in the absence of sophisticated haemodynamic monitoring or readily available markers of end-organ perfusion. This may be the most urgent area for clinical research.
