# Correction: Feasibility of Modified Surviving Sepsis Campaign Guidelines in a Resource-Restricted Setting Based on a Cohort Study of Severe *S. Aureus* Sepsis

**DOI:** 10.1371/annotation/7f498e31-2709-44f6-877a-d0ee89bebe03

**Published:** 2012-05-31

**Authors:** Weera Mahavanakul, Emma K. Nickerson, Pramot Srisomang, Prapit Teparrukkul, Pichet Lorvinitnun, Mingkwan Wongyingsinn, Wirongrong Chierakul, Maliwan Hongsuwan, T. Eoin West, Nicholas P. Day, Direk Limmathurotsakul, Sharon J. Peacock

The word "S. aureus" was formatted incorrectly. The correct title is: Feasibility of Modified Surviving Sepsis Campaign Guidelines in a Resource-Restricted Setting Based on a Cohort Study of Severe *S. aureus* Sepsis. The correct citation is: Mahavanakul W, Nickerson EK, Srisomang P, Teparrukkul P, Lorvinitnun P, et al. (2012) Feasibility of Modified Surviving Sepsis Campaign Guidelines in a Resource-Restricted Setting Based on a Cohort Study of Severe *S. aureus* Sepsis. PLoS ONE 7(2): e29858. doi:10.1371/journal.pone.0029858

